# Hemolysis Precedes Urine Color Change in Patients Undergoing Open-Heart Surgery on Cardiopulmonary Bypass

**DOI:** 10.7759/cureus.49971

**Published:** 2023-12-05

**Authors:** Kenta Takeishi, Suguru Yamamoto, Yasuto Sato, Osamu Takahashi, Nobuko Fujita, Kohei Abe, Yoko Fukui, Megumi Kameda, Tokujiro Uchida, Yasuko Nagasaka

**Affiliations:** 1 Department of Anesthesiology, St. Luke's International Hospital, Tokyo, JPN; 2 Department of Anesthesiology, Graduate School of Medical and Dental Sciences, Tokyo Medical and Dental University, Tokyo, JPN; 3 Department of Anesthesiology, School of Medicine, Tokyo Women’s Medical University Hospital, Tokyo Women’s Medical University, Tokyo, JPN; 4 Graduate School of Public Health, Shizuoka Graduate University of Public Health, Shizuoka, JPN; 5 Graduate School of Public Health, St. Luke's International University, Tokyo, JPN; 6 Department of Cardiovascular Surgery, St. Luke’s International Hospital, Tokyo, JPN; 7 School of Medicine, Tokyo Women’s Medical University Hospital, Tokyo Women’s Medical University, Tokyo, JPN

**Keywords:** luminance contrast analysis, cardiac surgery, acute kidney injury, urine color change, cardiopulmonary bypass, hematuria, hemolysis

## Abstract

Purpose: Red-colored urine often occurs in patients in the perioperative period who undergo cardiac surgery using cardiopulmonary bypass (CPB). This urine color change has been utilized for approximating hemolysis during CPB without a proven relationship for ongoing hemolysis. This case series study aimed to examine the relationship between plasma free hemoglobin (Hb) levels and quantified measures of urine color.

Methods: Ten patients were enrolled in this study. Blood and urine were collected for analyses for the following time points: before surgery, two hours after the initiation of CPB, every 30 min during CPB thereafter, and 0, 2, 4, 12, and 24 hours after the completion of CPB. We measured free Hb in plasma and urine using the azide-methemoglobin method. Photographs of urine were obtained, and the luminance of the three basic colors (red/green/blue) was analyzed by quantitative luminance contrast analysis to find a correlation for hemolysis.

Results: Median levels of plasma free Hb were 0.015 (0.010-0.080, n = 10) g/dL at baseline. During the CPB, increases in plasma free Hb levels were measured: median plasma free Hb levels were increased to 0.100 g/dL (0.020-0.240, p = 0.039, vs. baseline, n = 9) at two hours into CPB, median and range, respectively. In contrast, increases in urinary free Hb levels and/or urine color changes were measured only after cessation of CPB in nine patients.

Conclusion: Urine color change or elevation of urinary free Hb levels followed the elevation of plasma free Hb levels with considerable delay.

## Introduction

Postoperative acute kidney injury (AKI) following open-heart surgery increases perioperative mortality [1,2], and up to 40% of patients undergoing major cardiovascular surgeries develop postoperative AKI [3,4]. Increasingly, evidence suggests prolonged periods of cardiopulmonary bypass (CPB) over 140 min increases the risk of postoperative AKI [5]. Multiple factors associated with CPB impact postoperative kidney function, namely, hemolysis, inflammation, oxidative stress, renal ischemia, reperfusion, cholesterol emboli, or toxins [6]. The detailed mechanisms by which hemolysis induces kidney dysfunction are unknown, but it is plausible that nitric oxide (NO) scavenging by plasma free hemoglobin (Hb) plays a significant role in the pathophysiology of postoperative AKI. In the setting of hemolysis, a lack of NO by scavenging [7] leads to a sequence of events, including platelet activation and aggregation, hemolysis-associated smooth muscle dystonia, vasculopathy, and endothelial dysfunction [8]. In addition, AKI induced by a lack of NO secondary to intravascular hemolysis may be explained via interruption of renal microcirculation [9].

It has been reported that elevated levels of plasma free Hb above 10 µmol/L (0.064 g/dL) at the end of prolonged (~180 min) exposure to the CPB circuit during open-heart surgeries correlate with postoperative AKI [10]. In human subjects, inhalation of NO in patients undergoing open-heart surgery on CPB prevented not only postoperative AKI but also stage III chronic kidney disease (CKD) [4].

Techniques to measure the degree of hemolysis reported in the literature, for example, established analyses for plasma free Hb levels (i.e., tetramethylbenzidine spectrophotometric [11] or azide-methemoglobin method [12]) are available. However, none of the measurements are without the withdrawal of blood samples or costs for measurements. On the other hand, red-colored urine may occur in patients during and after CPB, which is often considered a sign of hemolysis [13,14]. Therefore, we hypothesized that quantification of urine color change may be an inexpensive and non-invasive alternative to determine the degree of hemolysis. In this study, we quantified hematuria by urine luminal transmittance technique and referred the results to concurrent measurement of plasma free Hb levels, in patients undergoing open-heart surgery on CPB.

The contents of this manuscript have been presented at the 2018 Annual Meeting of the American Society of Anesthesiologists (A1166, October 13, 2018).

## Materials and methods

This case series analysis study was conducted after the approval of the Institutional Review Board at St. Luke’s International Hospital, Tokyo, Japan (#16-R115), and written informed consent was obtained from each patient.

The primary endpoint of this study was to describe whether hematuria, either grossly determined or measured by urine color luminal transmittance analysis, reflects ongoing elevations of plasma free Hb levels. We included patients scheduled for cardiac or aortic surgery using CPB, with an estimated CPB duration of more than two hours. Because we aimed to describe the degree of hematuria associated with ongoing hemolysis, patients who were already notable for comorbidities associated with hemolysis, such as hemoglobinopathies (i.e., sickle cell disease), paroxysmal nocturnal hematuria, vasculitis, malignancies, sepsis, those who already were on renal replacement therapy, those who received blood transfusions within one week, or those who were hemodynamically unstable, were excluded.

Anesthesia

General anesthesia was induced by propofol and fentanyl with or without midazolam. After muscle relaxant administration of rocuronium, patients were intubated and ventilated. Sevoflurane, a volatile anesthetic and intravenous propofol, and opioids such as fentanyl and remifentanil were given throughout the procedure to maintain anesthesia.

Sample collections

**Figure 1:**
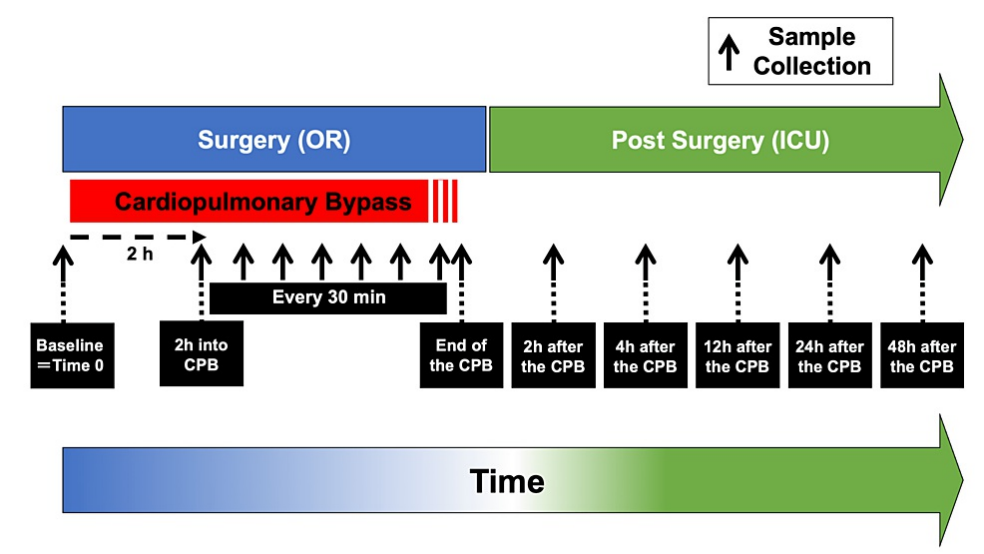
Scheme of plasma and urine sampling schedule Whole blood and urine samples were obtained at pre-CPB (before surgery), two hours after the initiation of CPB, every 30 min thereafter, at the cessation of CPB (protamine administration), 2, 4, 12, 24, and 48 hours after the completion of CPB.

Whole blood (2 mL) was withdrawn from the radial artery catheter at a speed no faster than 1 mL per second and was poured into a vacutainer tube (EDTA-2K # 367846; Becton Dickenson Japan, Tokyo, Japan). To avoid mechanical hemolysis, we removed the cap to release negative pressure and gently poured the blood into the tube. Urine from the transurethral bladder catheter was collected into a urinary meter bag (#153204; Bard Medical, Becton and Dickinson, NJ, U.S.A), and its meter canister was emptied every 30 min and just before the urine collection. Fresh urine samples were separated into a transparent tube at each time point (10 mL, #EK12 tube, Eiken Chemical Co., Ltd, Tokyo, Japan), and photographs were taken. Blood and urine samples were obtained at the following timing: baseline (pre-CPB), two hours after the initiation of CPB, and every 30 min thereafter until the completion of CPB (Figure 1).

Imaging and color analysis of urine samples

**Figure 2:**
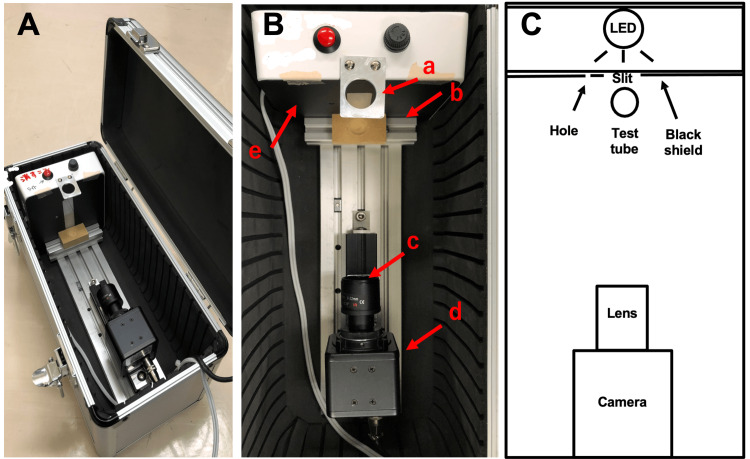
Configuration of the camera box Description of the camera box, developed to obtain photographs for the macroscopic color measurements of the urine samples. A: Camera system in a shielded box B: View from above a. LED light box, b. Tube stand, c. Camera lens, d. Camera body, e. Black shield C: Scheme of the view from above

**Figure 3:**
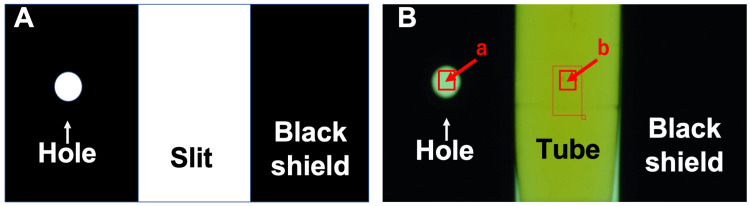
Representative photograph of a test tube A. Picture of a test tube (b) with an additional small margin area (a) as a baseline. B. Black shield with a hole for baseline calibration a. Additional margin area as a baseline b. Picture

To quantify urine color as a computable measure, images of fresh urine after centrifugation (4 °C, 2,500 rpm for 10 min) were taken by a high-resolution camera (CMOS camera; ELP, ELP-USBFHD01M-MF80, 5MPixel CCTV lens 6-22 mm with IR cut) in a light-tight sealed box (32 x 13 x 12 cm; Figure 2, Panels A, B, and C). A test tube without urine (for transmitted radiant flux) or with urine was set up in the sealed box, and a white LED light was illuminated behind the black shield (dimensions: 11.5 cm x 11.5 cm; slit: 1.5 cm x 2.0 cm; hole: 4 mm in diameter; 2 cm apart from the slit; Figure 3, Panel A) while images were taken. Images (Figure 3, Panel B) were analyzed for transmittance of the three basic colors, red (R), green (G), and blue (B), by a luminance contrast method (Aeternus; Aeternus Color Analyzer).

Analyzing color using the L, a, and b axes is the standard technique in the optical field [15]. However, we chose the green/red/blue analysis because it is visually understandable. To the best of our knowledge, there is no such clinical study that examines samples using LED transmittance.

Because green is a complementary color of red, we adopted green color as a pre-set background color to enhance color change by hemolysis. The transmittance of each color was calculated by the following equation:

Transmittance = (X of a sample at a certain time point) / (Y of the empty tube as transmitted radiant flux) X: brightness of R, G, or B of the tube containing plasma or urine sample Y: brightness of R, G, B of an empty tube

Free Hb-level measurements of plasma and urine

Whole blood and urine were centrifuged at 4 °C, 2,500 rpm for 10 min, and free Hb levels of plasma and urine were measured by HemoCue®Plasma/Low Hb system (HemoCue®; HemoCue AB, Ängelholm, Sweden) [16], a hemoglobinometer that uses the azide-methemoglobin method. HemoCue® converts plasma or urinary free Hb to methemoglobin by sodium nitrite, which forms a complex with azide to produce azide-methemoglobin. The machine utilizes two transmittance wave strengths, 570 nm, and 880 nm, to determine the azide-methemoglobin quantity in µmol per liter (µmol/L). HemoCue's measurement of free hemoglobin levels in biological fluids, to known hemoglobin levels, are linear between 0.03 and 3.00 g/dL, according to the manufacturer’s instructions [16]. To avoid errors, each sample was subjected to duplicate measurements, and a mean value was analyzed.

Statistical analysis

Statistical analyses were performed with GraphPad Prism 8 (GraphPad Software, Inc., San Diego, CA). We conducted the Wilcoxon signed rank test to compare the baseline and the levels of plasma Hb at two hours into the CPB, where an apparent increase in plasma free Hb was measured.

Considering the small sample size and the distribution of plasma free Hb, we conducted non-parametric tests in this study. Of note, non-parametric tests are more conservative than parametric tests, making them less likely to obtain statistical significance.

## Results

Patient characteristics

Ten patients (eight males and two females) were enrolled in this study. Types of surgeries include aortic (n = 3), valvular repair (n = 4), a combination of coronary artery bypass grafting and valvular repair (n = 1), and a combination of valvular repair and aortic surgery (n = 2). In particular, four patients underwent surgery using deep hypothermic circulatory arrest. Preoperative CKD by the KDIGO criteria (GFR categories in chronic kidney disease G3a and above; GFR < 60 mL/min) was present in six patients (Patients #1, #2, #5, #6, #8, and #9). There was no postoperative CKD stage progression during the study period. Postoperative AKI by the KDIGO criteria (an absolute 0.3 mg/dL rise of serum creatinine within 48 hours or 1.5-fold rise within seven days) was present in two patients (Patients #1 and #3). General anaesthesia was maintained with volatile anaesthetics (sevoflurane, n = 9) or total intravenous anaesthesia with propofol (n = 1). Allogenic blood was given to the patients who received blood transfusion (n = 6), except for Patient #4, who received autologous blood. The median CPB time was 186 min (171.5-231.2) (Table 1). A centrifugal pump (COBE® RevolutionTM Centrifugal Blood Pump; LivaNova, London, UK) was utilized in all patients. However, types of CPB cannula and circuits differed between the patients (Table 2).

**Table 1:** Baseline clinical valuables and procedural characteristics Age, sex, BMI, renal function (eGFR at the baseline and 30 days after surgery, AKI), type of surgery, surgery/CPB time, blood loss, amount of urine, red cell transfusion, and use of IABP/circulatory arrest are shown. Abbreviations: BMI, body mass index; eGFR, estimated glomerular filtration rate; IABP, intra-aortic balloon pumping; CSF, cerebrospinal fluid; MAP, mitral annuloplasty; TAP, tricuspid annuloplasty; LAA, left atrial appendage; AVP, aortic valvuloplasty; MVP, mitral valve plasty; CABG, coronary artery bypass grafting

Pt	Age (yo)	Sex	BMI (kg/m^2^)	eGFR (ml/min/1.73m^2^)		Post Surgery AKI（KDIGO）	Procedure	Surgery time (min)	CPB time (min)	Blood loss (ml)	Urine (ml)	Red cell transfusion (ml)	IABP	Circulatory arrest
				Baseline	30 days after surgery									
#1	71	M	20.6	20.4	22.9	Yes	Graft replacement of descending thoracic aorta	472	239	2401	130	2240	No	Yes
															#2	48	M	33	30.3	35.5	No	Graft replacement of thoracoabdominal aorta, CSF drainage	318	169	1905	970	0	No	Yes
#3	73	M	23.4	65.9	71.3	Yes	MAP, TAP, maze procedure, LAA closure	462	243	2605	410	280	No	No															
															#4	24	F	24.4	137.9	145	No	AVP, graft replacement of ascending aorta	301	119	1068	270	0	No	No
#5	72	M	20.8	41.6	43.4	No	AVP, CABG	379	190	1021	905	1120	No	No															
															#6	86	M	22.8	57.8	58.5	No	Graft replacement of arch to descending thoracic aorta, MAP, maze	319	179	1272	1015	1120	Yes	Yes
#7	87	M	24	68.1	72.9	No	AVP, MAP, TAP, LAA closure	333	182	685	627	0	No	No															
															#8	64	M	24.2	50	49.1	No	David procedure	375	208	1137	1895	0	No	No
#9	79	F	17.4	54.5	57.7	No	AVR, MVP	298	152	185	810	840	No	No															
															#10	76	M	23.5	73.5	91.2	No	Total arch replacement + CABG	494	304	996	1613	560	Yes	Yes

**Table 2:** Detailed type of pump, cannulae, and time of CPB A centrifugal pump was utilized in all patients. Type of cannula, circuit (i.e., use of cerebral perfusion), and use of circulatory arrest are different between patients due to the type of surgery.

Pt	CPB time (min)	Circulatory arrest	Circulatory arrest time (min)	Pump type	Cerebral perfusion	Cerebral perfusion time (min)	Aortic cannula	Venous cannula
#1	239	Yes	82	centrifugal	No		FEMII016AS Edwards, EZ Glide cannula 21Fr Edwards	QuickDraw 25Fr Edwards, DLPMalleable 28Fr Medtronic
#2	169	Yes	23	centrifugal	Yes	10	FEM II 16AS Edwards, FEM II 20AS Edwards	QuickDraw 25Fr Edwards, DLP Malleable 28Fr Medtronic
#3	243	No		centrifugal	No		EZ Glide cannula 21Fr Edwards	DLP Malleable 28Fr Medtronic, Straight 32Fr Edwards
#4	119	No		centrifugal	No		EZ Glide cannula 21Fr Edwards	DLP Pacifico 29/29Fr Medtronic
#5	190	No		centrifugal	No		EZ Glide cannula 21Fr Edwards	DLP Pacifico 29/29Fr Medtronic
#6	179	Yes	16	centrifugal	Yes	22	EZ Glide cannula 21Fr Edwards	DLP Malleable 28Fr Medtronic, Straight 32Fr Edwards
#7	182	No		centrifugal	No		EZ Glide cannula 21Fr Edwards	DLP Malleable 28Fr Medtronic, Straight 32Fr Edwards
#8	208	No		centrifugal	No		EZ Glide cannula 21Fr Edwards	VC2 Atrial Caval cannulae 34/48 Medtronic
#9	152	No		centrifugal	No		EZ Glide cannula 21Fr Edwards	DLP Malleable 28Fr Medtronic, Straight 32Fr Edwards
#10	304	Yes	38	centrifugal	Yes	66	EZ Glide cannula 21Fr Edwards	VC2 Atrial Caval cannulae 34/48 Medtronic

Plasma free Hb levels

**Figure 4:**
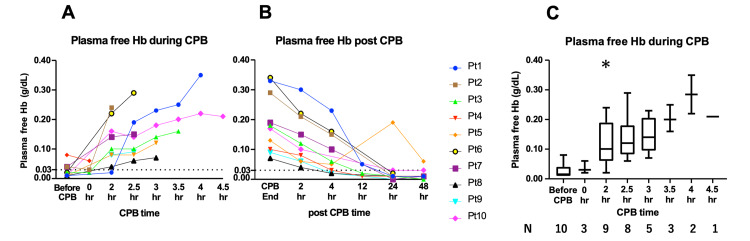
Plasma free Hb levels during and following the CPB Plasma free Hb levels were measured during (A) and after (B) the CPB in each patient. Except for Patient #1, every plasma presented with elevated (>0.03 g/dL) free Hb levels at two hours into CPB. At the end of the CPB, the levels reached maximum and then decreased to the baseline at 48 hours after the CPB. Statistical analysis of plasma free Hb levels during CPB (C). Plasma free Hb significantly differed from baseline at two hours. The data are shown as median and minimum to maximum * p < 0.05, vs baseline, compared with the nonparametric Wilcoxon signed rank test. The significance level was set at 5%(C). N numbers: Baseline (n=10), 0 h into CPB (n=3), 2 h into CPB (n=9), 2.5 h into CPB (n=8), 3 h into CPB (n=5), 3.5 h into CPB (n=3), 4 h into CPB (n=2), 4.5 h into CPB (n=1).

Every patient's plasma free Hb levels increased toward the end of CPB (Figure 4A). The median baseline plasma free Hb levels were 0.015 g/dL (range: 0.010-0.080, n = 10). During the CPB at two hours after the initiation, plasma free Hb levels reached 0.100 g/dL (0.020-0.240, p = 0.039, vs., baseline, n = 9), median and range, respectively (Figures 4A, 4C). After the CPB, plasma free Hb of all the patients, except Patient #5, returned to the baseline levels within 24 h (Figure 4B).

Urinary free Hb levels

**Figure 5:**
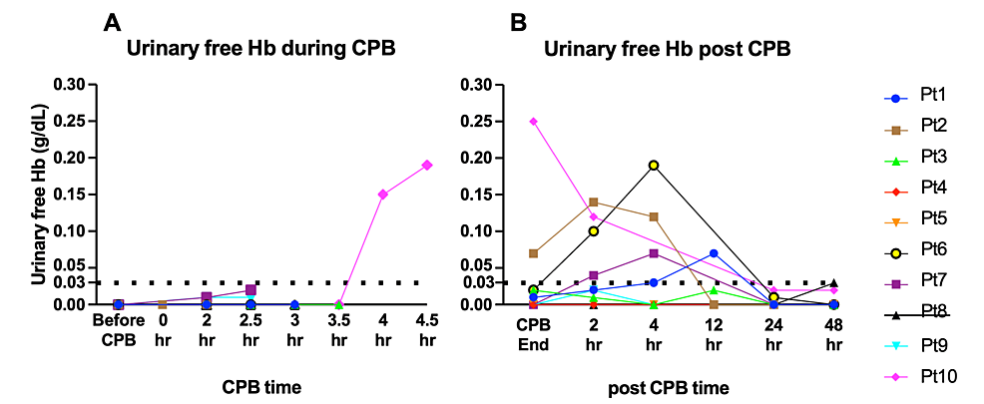
Urine free Hb levels during and following the CPB The urine free Hb levels were measured during (5A) and after (5B) the CPB in each patient. Elevated levels of urinary free Hb were only measured in Patient #10 during CPB. In Patients #1, #2, #6, #7, and #10, urinary Hb levels transiently increased above 0.03 and returned to the level below 0.03 after CPB.

The urinary free Hb levels varied between the individuals. However, no patient presented urinary free Hb levels above the accuracy range of HemoCue® Plasma/Low Hb photometer (i.e., >0.03 g/dL), up to 3.5 h into CPB (Figure 5). Patient #10 was the only patient who received >3.5 hour duration of CPB, up to 304 minutes, and presented hematuria at four and 4.5 hours into CPB. Of note, Patient #10’s past medical history was notable for benign prostate hypertrophy. In Patients #1, #2, #6, and #7, the urinary free Hb levels transiently and mildly increased after CPB.

Macroscopic findings of the urine color

**Figure 6:**
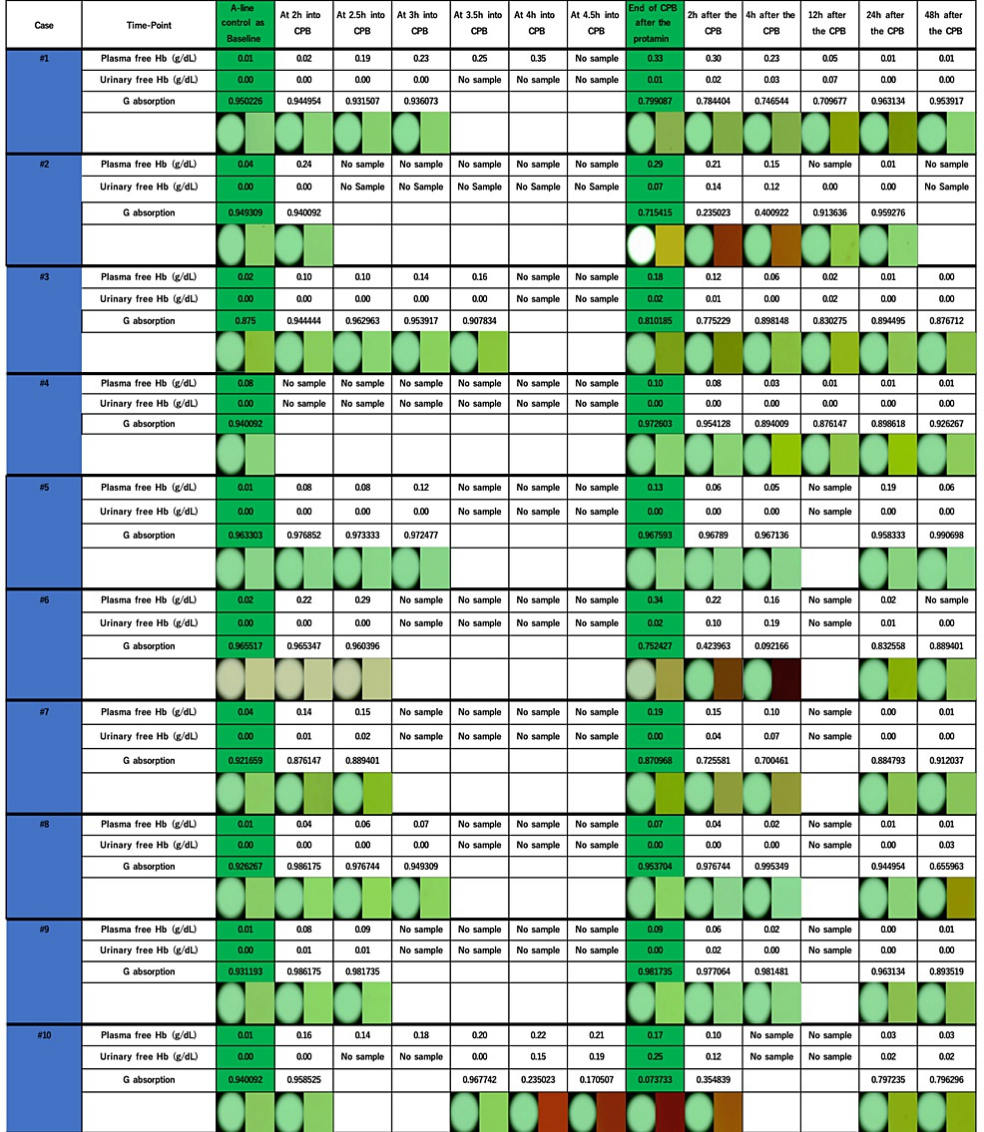
Photographs of urine and free Hb levels of urine and plasma of each patient Plasma free Hb, urinary free Hb, green light transmittance, and macroscopic finding of the urine samples. Plasma and urine photographs were taken with a preset green background color to enhance color change by hemolysis, and each control baseline color level was subtracted from the color analysis.

Macroscopic appearances of each urine sample were straw at baseline. After commencing CPB, the tinge of red urine color began at different time points and degrees between patients (Figure 6). Urine color abruptly changed in Patients #2, #6, and #10; in contrast, changes in urine color were subtle in Patients #1, #3, and #7. All the urine colors returned to the baseline levels by the end of the study period (~48 hours after the cessation of CPB), except for Patient #8.

The third image of Patient #3 and the first four images of Patient #6 were obtained under a different background setting. Although with different background colors, changes of color were detected by luminal transmittance (sample color minus background color under the identical background setting per each sample), similar to other samples measured at the equivalent time points (see luminal transmittance analysis of the urine color section).

Luminal transmittance analysis of the urine color

**Figure 7:**
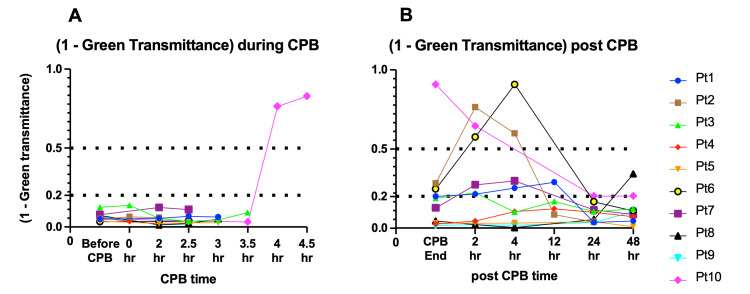
Urine color analyses by urinary green transmittance Urine green transmittance was measured during (A) and after (B) the CPB in each patient. In every patient, except for Patient #10, changes in green light transmittance were almost negligible during the CPB (A). After the CPB, in Patients #2, #6, and #10, green transmittance transitioned below 0.5, and in Patients #1, #3, #8, and #7, green transmittance converted between 0.5 and 0.8.

Urine color change was analyzed with green, blue, and red-light transmittance. Because the green color complements red (i.e., red is the color of plasma with hemolysis), green was the most sensitive to detect the hemolytic change of the urine samples. Thus, the severity of hematuria should correlate with decreased transmittance of green light (Figure 7).

Changes in green light transmittance were almost negligible during the CPB, except for Patient #10, who underwent the longest (304 min) duration of CPB. After the cessation of individually different durations of CPB, there were a variety of changes in the transmittance of urine samples between patients.

In Patients #2, #6, and #10, green light transmittance levels gradually returned to baseline once they decreased to above 0.5. On the other hand, urinary green transmittance gradually reduced after the end of CPB and returned to the baseline in Patients #1, #3, and #7. Additionally, in Patient #8, it declined 48 hours after the CPB. In these patients (Patients #1, #3, #7, and #8), it decreased to a level between 0.5 and 0.8. In the other patients (Patients #4, #5, and #9), it showed only little changes at around the end of CPB. The change in red light transmittance was small, and the change in blue light transmittance was respective among patients.

## Discussion

In this study, we report that, in patients undergoing open-heart surgery, 1) the progress of CPB subsequently increased plasma free Hb levels; 2) elevated plasma free Hb levels returned to baseline within 48 hours after the cessation of CPB; and 3) during the CPB, urinary free Hb levels or urine color change only appeared at four hours after commencing the CPB. These results indicate that urinary excretion of free Hb was delayed a while after the elevations of plasma free hemoglobin levels.

Urine free Hb measurements have been the traditional standard procedure for detecting hemolysis in patients undergoing CPB. However, in our study, urinary free Hb levels did not increase in half of the patients regardless of ongoing hemolysis, and the other half increased to the detectable level, far later than plasma free Hb levels that reached the maximum levels. These results were in accordance with Windsant et al.’s study, which could not detect increases in urine free Hb levels in patients undergoing prolonged periods of CPB (163 ± 56 min) [10].

Even though mild/moderate urine color changes were measured in Patients #1, #3, #7, and #8 according to urine color change (green transmittance and visual change), there were patients with undetectable (Patients #3 and #8) or elevated (Patients #1 and #7) urinary free Hb levels. This observation implies that urine color analysis is more sensitive to detect hemolysis than urinary free Hb measurements by HemoCue. Moreover, although there were no or little urine hemolyses (either changes in green light transmittance or elevation of free Hb levels in urine) in Patients #4, #5, and #9, their maximum plasma free Hb levels were above 0.03 g/dL. Every patient with severe hemolysis (Patients #2, #6, and #10) underwent deep hypothermic cardiac arrest, a procedure known to be associated with hemolysis [17]. Additionally, Patients #6 and #10 underwent surgery using intra-aortic balloon pumping (IABP), which is a mechanical device that induces hemolysis [18].

In the current study, urinary transmittance analysis could more keenly detect urine free Hb, vs. urine free Hb-level measurements. Thus, urine color analysis may enable clinicians to promptly quantify the degree of hematuria while patients are on CPB. However, urine color changes did not reflect the timely diagnosis of the elevations of plasma free Hb levels during CPB. In detail, in Patients #4, #5, and #9, visual urine color change or color analyses were unchanged, and urine free Hb stayed below the measurement accuracy range; at that time, plasma free Hb levels were already elevated. Of note, a number of studies suggest that, in patients undergoing open-heart surgery, plasma free Hb levels above 0.06 g/dl are a possible threshold for postoperative acute kidney failure [6,10,19]. Not only the actual values of plasma free Hb levels, the free Hb ratio may also be a nuevo and reliable measure to predict AKI after CPB, which compares the levels of plasma free Hb, at the end of CPB divided by the baseline [20]. Therefore, in current medicine, it may be critical to determine plasma free Hb levels rather than to pursue urinary free Hb levels or urine color change relentlessly to initiate actions to prevent postoperative AKIs.

We measured free Hb levels by HemoCue®, which utilizes the azide-methemoglobin method, which was initially described by Vanzetti in 1966 [21]. The device uses a chemically covered cuvette and dry reagents, such as sodium deoxycholate, sodium nitrite, and sodium nitrite deposited on the inner wall of the cuvette cavity to lyse the red cells and convert Hb into azide-methemoglobin [22]. HemoCue® calculates Hb using spectrophotometric analysis and the transmittance of Hb peaks at 565 and 880 nm by using a dual-wavelength photometer. According to the manufacturer, this measurement system is linear to known Hb levels in biological fluids, within the range of 0.03-3.00 g/dL [22]. Because the HemoCue cuvette may be costly and not all clinicians can possess this device, we sought to determine the analysis of urinary photographs in the current study, according to other reports [23]. We assembled a sealed camera system to detect subtle urinary color changes. It cost less than 100,000 yen (≒680 dollars as of November 2023). We did not use complicated machines or software, and RBG analysis is the standard way to analyze color performed elsewhere. Moreover, to effectively capture the urine color change from straw (non-hemolysis) to red (hemolysis), we used background green color light in all patients to enhance its sensitivity. This system was able to detect hematuria by decreasing green transmittance in the urine of patients with severe hemolysis (Patients #2, #6, and #10), which at the same time urine free Hb levels increased. HemoCue® also uses transmittance, but the most significant difference between measurements of direct green transmittance and HemoCue® is that HemoCue® indirectly depicts free Hb levels by using the known correlation of free hemoglobin levels and its transmittance.

There are some limitations in this study. First, this is a descriptive single-center study of 10 patients. Given the fact that hemolysis by CPB differs largely between the institutions or the surgeons, our results may not apply to patients receiving heart surgeries on CPB in different settings. Even though this study is from one hospital, the degree of injury according to the types of surgical procedures and patients’ backgrounds in this study resulted in being diverse. Second, CPB time differed between the patients, making it difficult to compare their plasma free Hb, urine free Hb, and urine color changes while analyzing the decay of each level. Third, urinary tract bleeding may cause false-positive hematuria to reflect hemolysis. Patient #10 was notable for the long duration of CPB time, and we observed significant hematuria at 4.5 hours into CPB, prompting him to develop hemolysis. On the other hand, his preoperative urinalysis was already positive for hematuria (occult blood 1+), and his past medical history was notable for benign prostate hypertrophy. It was plausible that his preoperative hematuria and/or mechanical insertion of the urinary catheter between the hypertrophic prostate may have further severed the hematuria. In fact, after the maximum period of CPB time among the participants, Patient #10 developed more significant hematuria than other patients, which lasted until at least 48 hours after CPB. Fourth, as we did not have any information to estimate the strength of the association of urinary contrast analysis and plasma free Hb, we did not perform sample size calculations before conducting this case series study of 10 patients. Fifth, the analyses presented here are exploratory rather than confirmatory, as the n number is small. Sixth, postoperative AKI did not occur in patients other than Patients #1 and #3, and there are patients with plasma free Hb levels as high as or higher than Patients #1 and #3 (e.g., #2, #6, #10). These patients did not develop AKI for unknown reasons, as we only included 10 patients in this study. Lastly, urine color change is affectable by other factors, such as bilirubin, urine dilution due to hydration, or color-contained agents such as carbazochrome sodium sulfonate; however, we usually recognize hemolysis as a macroscopic color change in daily clinical practice. Our goal for this study was to establish a quantitative threshold that indicates hemolysis by analyzing urine. However, at the time that we could detect urine color change, plasma free Hb was already above the levels that have a risk of occurring postoperative AKI (0.03 g/dL). Thus, although we cannot apply our technique in a clinical setting, our results show that gross hematuria (or significant urine color change) is no longer a sensitive way to see hemolysis.

## Conclusions

In summary, this case series study reports plasma free Hb continued to increase during the CPB time, whereas hematuria remained unmeasurable in most of the patients. We confirmed that blood hemolysis precedes macroscopic hematuria for two to three hours in patients undergoing open-heart surgery on CPB. Therefore, we conclude that urine color change is not an incisive indicator for the early detection of hemolysis. Ongoing studies may excavate new biomarkers that may serve as crucial indicators for postoperative AKI in the future.

Larger human studies are warranted to determine the correlation between hemolysis and hematuria in patients undergoing open-heart surgery on CPB.
